# Measurement of T1 of the Ultrashort T2* Components in White Matter of the Brain at 3T

**DOI:** 10.1371/journal.pone.0103296

**Published:** 2014-08-05

**Authors:** Jiang Du, Vipul Sheth, Qun He, Michael Carl, Jun Chen, Jody Corey-Bloom, Graeme M. Bydder

**Affiliations:** 1 Department of Radiology, University of California San Diego, San Diego, California, United States of America; 2 Global Applied Science Laboratory, GE Healthcare, San Diego, California, United States of America; 3 Department of Neurosciences, University of California San Diego, San Diego, California, United States of America; University of Alberta, Canada

## Abstract

Recent research demonstrates that white matter of the brain contains not only long T2 components, but a minority of ultrashort T2* components. Adiabatic inversion recovery prepared dual echo ultrashort echo time (IR-dUTE) sequences can be used to selectively image the ultrashort T2* components in white matter of the brain using a clinical whole body scanner. The T2*s of the ultrashort T2* components can be quantified using mono-exponential decay fitting of the IR-dUTE signal at a series of different TEs. However, accurate T1 measurement of the ultrashort T2* components is technically challenging. Efficient suppression of the signal from the majority of long T2 components is essential for robust T1 measurement. In this paper we describe a novel approach to this problem based on the use of IR-dUTE data acquisitions with different TR and TI combinations to selectively detect the signal recovery of the ultrashort T2* components. Exponential recovery curve fitting provides efficient T1 estimation, with minimized contamination from the majority of long T2 components. A rubber phantom and a piece of bovine cortical bone were used for validation of this approach. Six healthy volunteers were studied. An averaged T2* of 0.32±0.09 ms, and a short mean T1 of 226±46 ms were demonstrated for the healthy volunteers at 3T.

## Introduction

Over one million people in the United States are affected by multiple sclerosis (MS), a disease that presumably affects myelin of the central nervous system [1]. Myelin is a lamellar membranous structure consisting of alternating protein and lipid layers [2]. It helps to increase nerve conduction velocity by insulating the axons from electrically charged atoms and molecules. The loss of myelin sheath is the hallmark of numerous inflammatory and neurodegenerative disorders, which affect the nervous system including MS [3].

Magnetic resonance imaging (MRI) plays an important role in diagnosing as well as monitoring progression and response to therapy in demyelinating diseases because of the high contrast between normal and abnormal tissue. Researchers have been working for decades to develop non-invasive MRI techniques to accurately characterize myelin in vivo [4]–[7]. The non-water protons in myelin have extremely short T2s and cannot be directly imaged with conventional clinical MRI sequences [8], [9]. The water associated with myelin of the central nervous system can be used to indirectly quantify myelin. This approach exploits the fact that myelin water (water trapped in the myelin sheath) has a much shorter T2 than axon water (water within myelinated axons) and mixed water (interstitial water) [4]–[7]. The myelin water fraction, which is defined as the ratio of the signal intensity of the shortest T2 component to that of the total, has been used to estimate myelin content [10]. Although several histopathylogy studies have confirmed the sensitivity of myelin water fraction to myelin content [10], [11], this approach has several drawbacks. First, it is based on a multiple component non-negative least-squares (NNLS) fit of the T2 decay of the MR signal acquired with Carr-Purcell-Meiboon-Gill (CPMG) sequences. Multi-component analysis is very sensitive to noise and is technically challenging for clinical imaging [12]. Second, conventional CPMG sequences on whole body clinical scanners have minimal echo times (TE) which are too long to detect signal from the non-water protons in myelin as well as water very tightly bound to myelin.

Direct imaging of myelin is likely to significantly improve specificity in evaluating myelination and demyelination [8], [9]. Several groups have investigated the MR properties of myelin. Broad-line proton spectroscopy studies showed that myelin water is in a liquid-crystalline state [13]. Multi-component fitting of spin echo decays of fixed human white matter samples has shown extremely short T2 values of ∼50 µs for myelin protons [14]. NMR spectrometer studies have found the ultrashort T2 signals (50 µs<T2<1 ms) in myelinated nerve [8], and a wide distribution of T2* values ranging from 8 µs to 26 ms in the spinal cord [9]. These studies suggest that myelin can be directly imaged with ultra short echo time (UTE) sequences using NMR spectrometers. Several other studies suggest that the ultrashort T2* components (probably from myelin) can be directly imaged using ultrashort echo time (UTE) sequences on whole body clinical scanners [15], [16].

However, characterizing the ultrashort T2* components, including measurement of T1, T2 and T2* requires further consideration. While T2* can be measured with techniques such as two-dimensional adiabatic inversion recovery preparation and dual echo UTE (2D IR-dUTE) acquisition at a series of TEs, techniques for robust T1 measurement are not so obvious. The purpose of the present study was to investigate a novel approach for robust T1 measurement of the ultrashort T2* components in white matter of the brain in healthy volunteers using a clinical 3T scanner.

## Methods

### Pulse Sequence

**Figure 1:**
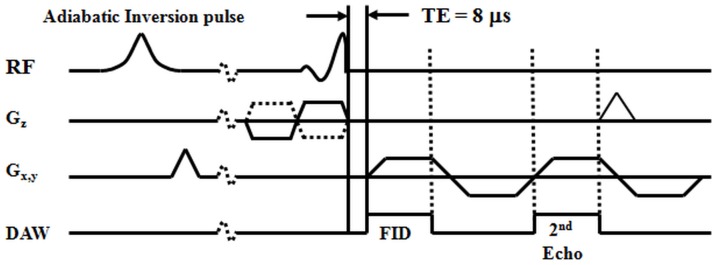
Pulse sequence for 2D IR-dUTE imaging of the ultrashort T2* components in white matter of the brain. A minimal nominal TE of 8 µs is achieved by using half pulse excitation and radial ramp sampling. An adiabatic inversion recovery preparation pulse is used together with dual echo acquisition to suppress the signal from the long T2 components in white matter and create high contrast for the ultrashort T2* components.

Figure 1 shows the basic 2D IR-dUTE sequence which was implemented on a 3T Signa TwinSpeed scanner (GE Healthcare Technologies, Milwaukee, WI). This whole body scanner has a maximum gradient strength of 40 mT/m and slew rate of 150 mT/m/ms. The sequence employed an adiabatic Silver-Hoult inversion radiofrequency (RF) pulse (duration  = 8.64 ms, bandwidth  = 1.4 kHz) which was used for long T2 signal suppression, and a half RF pulse (duration  = 472 µs, pulse bandwidth  = 2.7 kHz) which was used for short T2 signal excitation. These were followed by 2D free induction decay (FID) data sampling with a minimal nominal TE of 8 µs [17]. The radial half projections were repeated through 360° to cover the whole of k-space. 2D UTE images were reconstructed by regridding the projection data onto Cartesian grids followed by fast Fourier transformation (FFT).

### Short T2 Contrast Mechanism

The dominant signal in white matter of the brain is from the long T2 components [15], [16]. The ultrashort T2* components only represent a small fraction (e.g.,<10%) of the total signal [16]. T2 saturation approaches, including those based on the use of a long duration 90° pulse followed by gradient dephasing, and dual echo acquisitions followed by echo subtraction, are sensitive to B1 and/or B0 inhomogeneity, and may not work well [18]. As a consequence the residual long T2 signal may be significantly higher than that of the ultrashort T2* signal components, thus significantly reducing short T2 contrast and quantification accuracy. In contrast, adiabatic inversion pulses have the excellent property of uniform inversion of the longitudinal magnetization of the long T2 tissues or tissue components when B1 is above a certain threshold [19]. UTE data acquisition starts at a delay time (TI) necessary for the inverted longitudinal magnetization of long T2 white matter to reach the null point. Uniform and complete suppression of long T2 white matter is anticipated assuming the same T1 for different long T2 components in white matter [5]. Other long T2 tissues or tissue components, such as the gray matter, CSF and fat have T1s that differ from that of long T2 white matter. They are inverted but only partially nulled by the adiabatic IR pulse. The longitudinal magnetization of the ultrashort T2* components cannot be inverted due to significant transverse relaxation during the long adiabatic inversion process, and is subsequently detected by the 2D UTE data acquisition. Subtraction of the 2^nd^ echo image which acquires signals mainly from the residual long T2 tissues from the 1^st^ image would provide selective imaging of the ultrashort T2* components in white matter of the brain.

### T2* Measurement

Long T2* signal contamination is a major source of error in accurate T2* quantification of the ultrashort T2* components, which have far lower signal intensities than these of the long T2* components in white matter of the brain. We employed IR-dUTE imaging to minimize long T2* contamination. T2* of the ultrashort T2* components in white matter of the brain was measured via exponential fitting of the IR-dUTE signal acquired at a series of TE delays using the following single exponential signal decay model:
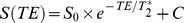
(1)where C accounts for the background noise, including pseudo-noise associated with undersampled IR-dUTE data acquisitions as well as residual long T2 signals.

### T1 Measurement

Quantification of T1 of the ultrashort T2* components in white matter of the brain requires the use of UTE sequences because of their extremely short T2*s. A primary inversion recovery based approach is not feasible due to the failure to invert the ultrashort T2* components using the pulses available on clinical scanners (which have limited RF power). Saturation recovery based approaches have been used for more accurate T1 measurement [20]. However, long T2* signal contamination is a major source of error. Regular saturation recovery UTE acquisitions may not provide accurate T1 estimations due to the basic problem of separating the signal from short T2* components from that from long T2* components (details discussed later).

Here we proposed an IR-dUTE based approach. With regular 2D UTE where the duration of the half RF pulses is of the order of T2*, the transverse magnetization for a steady-state UTE acquisition can be written as [17], [20]:
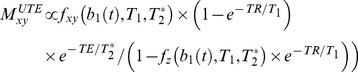
(2)where f_xy_ and f_z_ describe the behavior of the transverse magnetization and longitudinal magnetization, respectively, as a function of the half pulse b1(t) as well as the T2* and T1 of the ultrashort T2* components.

Recent studies demonstrate that the short T2 magnetization is partially inverted or saturated by the long adiabatic inversion pulse, and recovers during TI, after which it is partly excited by the half RF pulse [17], [21]. The longitudinal magnetization M_z_(T2) after the adiabatic IR pulse, neglecting T1, is approximately [19]:
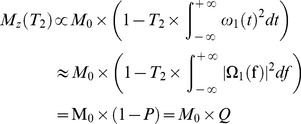
(3)


Here P is the attenuation factor due to long adiabatic IR pulse, and Q is the fraction of longitudinal magnetization following the adiabatic IR pulse.

The steady state transverse magnetization following an adiabatic IR pulse and a half RF excitation pulse can be calculated as follows [17]:
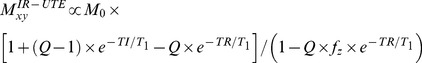
(4)


Our previous studies suggest that the ultrashort T2* components have T2*s around 0.3–0.5 ms, similar to those of cortical bone [16], [17]. Bloch equation simulation was performed based on Eq. 3, and it was found that Q was approximately 0 (<0.05 with a T2* of 0.4 ms), suggesting that the adiabatic IR pulse with a duration of 8.64 ms and a spectral bandwidth of 1.4 kHz almost completely saturates the longitudinal magnetization of the ultrashort T2* components in white matter of the brain. As a result, Eq. 4 can be simplified as follows:
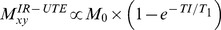
(5)


Eq. 5 suggests that T1 of the ultrashort T2* components can be reliably measured via exponential fitting of IR-dUTE images acquired with different combinations of TR and TI, on the condition that all these TR/TI combinations satisfy the nulling of long T2 components in white matter of the brain.

Because the ultrashort T2* components in the white matter of the brain have a very low proton density, the IR-dUTE signal is relatively low. Noise correction is important for accurate estimation of T2*, and especially T1. We used the approach proposed by Miller et al to correct the biased signal [22], namely:
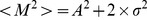
(6)where <M^2^> is the measured signal, A is the true signal amplitude which will be used for T2* and T1 quantification and σ is the background noise.

### Phantom and Specimen Study

A piece of rubber (Pink Eraser, Paper Mate Products Inc) was used for validation of the T1 and T2* measurements. A piece of fresh bovine cortical bone sample with a cross sectional thickness of 3 mm was harvested from tibial midshaft bought from a local slaughter house (Talone's Custom Slaughter House, 559 N Hale Ave, Escondido, CA). T1 validation was further performed on this bovine bone sample. Since conventional 2D slice selective UTE imaging is subject to eddy currents which may distort the slice profile and produce inaccurate T2* measurements [23], we modified the 2D UTE sequence by replacing the half pulse with a short rectangular pulse (duration  = 32 µs). The non-slice selective 2D UTE sequence was used to eliminate the eddy currents associated with the use of gradients in conventional slice selective half-pulse excitation. This also sped up data acquisition since only one excitation is needed for non-selective excitation compared with two excitations which are required for slice-selective excitation. The T2*s of the rubber and bovine cortical bone were measured using exponential fitting of the non-selective 2D UTE acquisitions obtained at a series of TEs (TE = 8, 100, 200, 400, 800, 1600, 3200 µs) based on Eq. 1. The T1s of the rubber and bovine cortical bone were also measured using non-selective saturation recovery 2D UTE acquisitions at a series of saturation recovery times (TSRs = 8, 25, 50, 100, 200, 400, 800 ms). The single exponential signal recovery model shown below was used to fit T1 [20]:
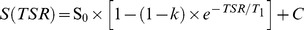
(7)where k accounts for the residual fraction of the longitudinal magnetization of cortical bone after a nominal 90° pulse. Other imaging parameters included: field of view (FOV) = 8 cm, acquisition matrix  = 128×128, number of half projections  = 403, sampling bandwidth  = 125 kHz, TR = 1000 ms, total scan time  = 47 minutes for both T2* and T1 quantification.

### Volunteer Study

In total six healthy volunteers (all male, age from 24 to 60 years, average age of 42±15 years) were recruited for this study. This study was reviewed and approved by the institutional review board (ethics committee) of the University of California, San Diego (UCSD), before the study began. And written informed consent approved by our Institutional Review Board was obtained prior to the participation of each subject. An eight-channel head coil was used for signal excitation and reception. The same rubber phantom was placed beside the brain for validation of in vivo T2* and T1 measurements. The following imaging parameters were used: FOV = 24 cm, slice thickness  = 5 mm, BW = 256 kHz, flip angle  = 70°, reconstruction matrix  = 256×256, number of projections  = 131, sampling points per projection  = 192. For T2* measurements, a TR of 1500 ms and a TI of 420 ms were used together with four sets of dual-echo UTE acquisitions (set #1: TE = 8 µs and 2.2 ms; set #2: TE = 0.1 and 2.2 ms; set #3: TE = 0.4 and 2.2 ms; set #4: TE = 2.2 and 4.4 ms). For T1 measurement the following four sets of TR/TI combinations: TR/TI = 750/260, 1000/320, 1500/420 and 2000/480 ms were used. The total scan time was 26 minutes for T2* measurement and 23 minutes for T1 measurement. The optimal TI was determined empirically by measuring the signal to noise ratio (SNR) of the white matter on the 2^nd^ echo image [16]. This should be zero or near zero since the ultrashort T2* components were expected to be decayed to zero or near zero when the long T2 components in white matter were effectively inverted and nulled by the long adiabatic IR pulse. Standard clinical sequences, including T1-weighted fast spin echo (FSE), T2-weighted FSE and proton-density (PD)-weighted FSE sequences, gradient recalled echo (GRE) sequence, T2-weighted fluid attenuated inversion recovery (T2-FLAIR) and magnetization prepared rapid acquisition gradient echo (MP-RAGE) sequences were also performed for comparison.

### Data Analysis

Quantitative evaluation of SNR, T2* and T1 were performed on each volunteer. SNRs were calculated as the ratio of the mean signal intensity inside a user drawn region of interest (ROI) to the standard deviation of the background noise (with the ROI drawn in air). T2* values were obtained using a Levenberg-Marquardt fitting algorithm based on Eq. 1 with the signal corrected based on Eq. 7. Saturation recovery based T1 measurements were also performed based on Eq. 7, while inversion recovery based T1 measurement was performed based on Eq. 5 with the signal corrected based on Eq. 7. The analysis algorithm was written in Matlab (The Mathworks Inc. Natick, MA, USA) and was executed offline on the DICOM images obtained using the protocols described above. After fitting was finished, goodness of fit statistics, including the R-squared value, mean squared error, root mean squared error and standard error were calculated.

## Results

**Figure 2:**
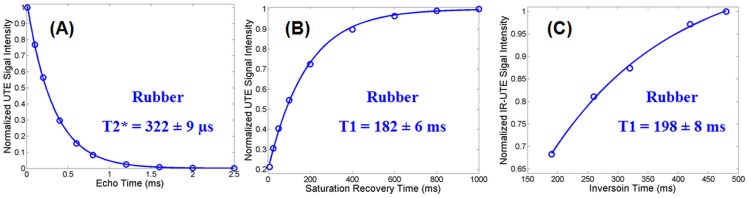
Non-selective 2D UTE measurement of T2* (A) and T1 (B, C) of a rubber phantom. A short T2* of 322±4 µs was measured by exponential decay curve fitting of UTE images at a series of different TEs (A). A short T1 of 182±6 ms was measured by exponential recovery curve fitting of saturation recovery UTE images acquired at a series of saturation recovery times (B). The novel IR-dUTE approach gave a short T1 of 198±8 ms (C), which is only a 3% overestimate relative to (B), suggesting that the new IR based technique is capable of accurate T1 measurement of short T2* species.

**Figure 3:**
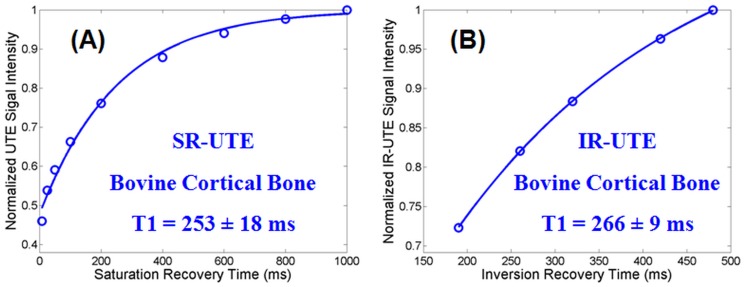
Non-selective 2D UTE measurement of T1 of a piece of bovine cortical bone: a short T1 of 253±18 ms was measured by exponential recovery curve fitting of saturation recovery UTE images acquired at a series of saturation recovery times (A). The novel IR-dUTE approach gave a short T1 of 266±9 ms (B), which is only a 5% overestimate relative to (A), further suggesting that the new IR based technique is capable of accurate T1 measurement of short T2* species such as cortical bone.

Figure 2 shows the T2* and T1 values for the rubber measured using the non-selective 2D UTE sequences. Excellent single component exponential decay and recovery curves were observed for the rubber which showed a short T2* of 322±4 µs and a short T1 of 182±6 ms with the saturation recovery approach as well as a short T1 of 198±8 ms with the IR approach. The new IR approach overestimated T1 by 8.8% relative to the T1 obtained with the saturation recovery UTE approach. Figure 3 shows measurement of T1 values for cortical bone using the non-selective 2D UTE sequences. The IR-UTE approach provided a T1 of 266±9 ms which is only a 5% overestimate relative to the T1 of 253±18 ms produced by the standard saturation recovery approach, further suggesting that the new IR based technique is capable of accurate T1 measurement of short T2* species such as cortical bone.

**Figure 4:**
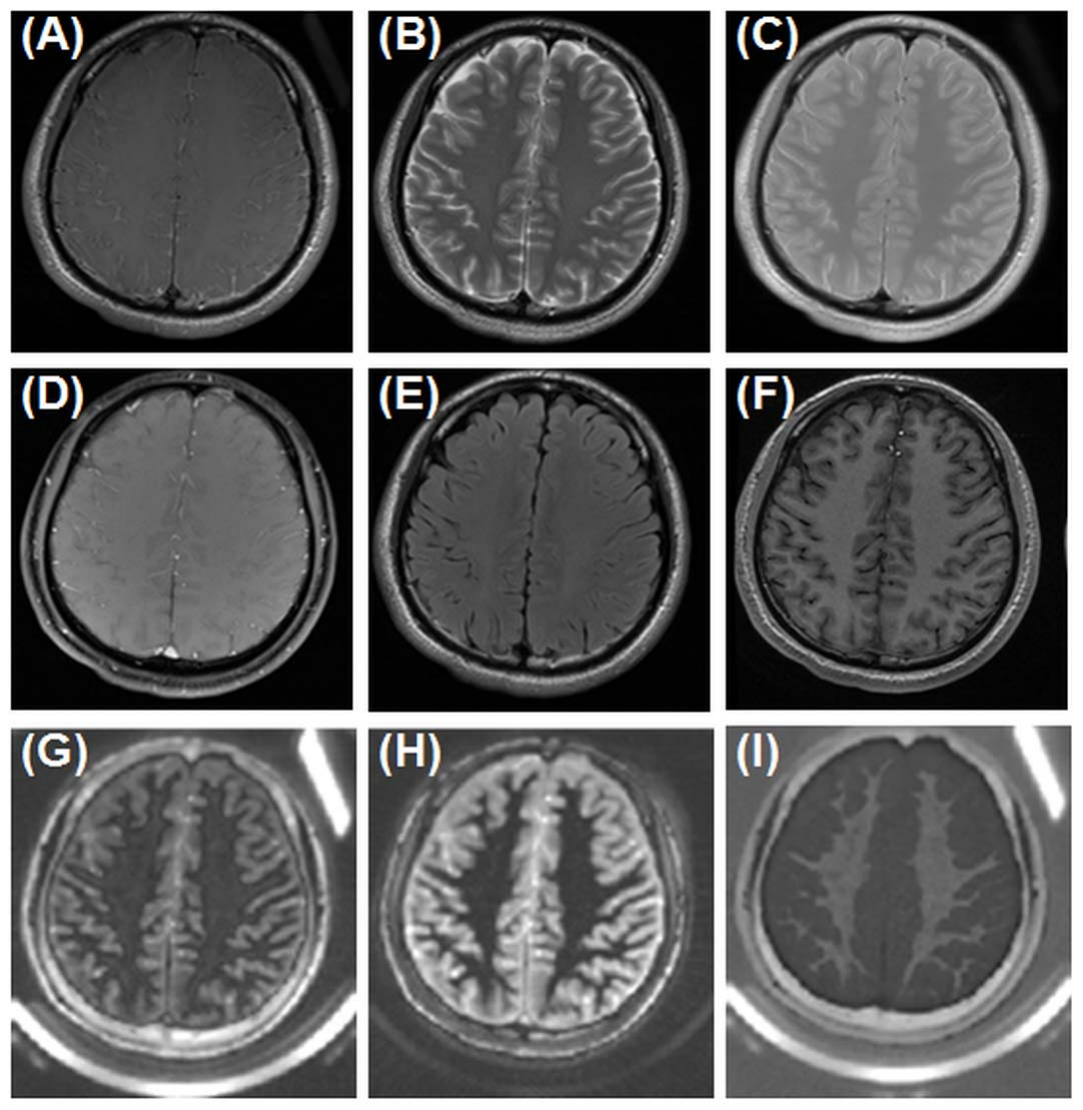
Conventional clinical imaging of the brain of a healthy volunteer using T1-FSE (A), T2-FSE (B), PD-FDE (C), GRE (D), T2-FLAIR (E), MP-RAGE (F), and IR-dUTE imaging with a TE of 8 µs (G) and 2.2 ms (H) as well as the corresponding echo subtraction image (I). The ultrashort T2* components in the white matter of the centrum semiovale appear hypointense in the 1^st^ echo image (G) but near zero signal in the 2^nd^ echo image (H), and are highlighted in the subtraction image (I). The rubber phantom is only seen with UTE based techniques.

Figure 4 shows a morphological comparison between IR-dUTE imaging and clinical T1-FSE, T2-FSE, PD-FSE, GRE, FLAIR and MP-RAGE imaging of white matter of the brain of a normal volunteer. Conventional clinical sequences with TEs of several milliseconds or longer showed signal predominantly from the long T2 components, with negligible signal from the short T2* component in white matter of the brain. The IR-dUTE sequence showed zero or near zero signal for the white matter on the 2^nd^ echo image with a TE of 2.2 ms (SNR∼2.6), directly confirming that the long T2 components in white matter were efficiently suppressed by the adiabatic inversion pulse so that only the ultrashort T2* components were detected by the IR-dUTE first echo image with a SNR of 17.2±3.3.

**Figure 5:**
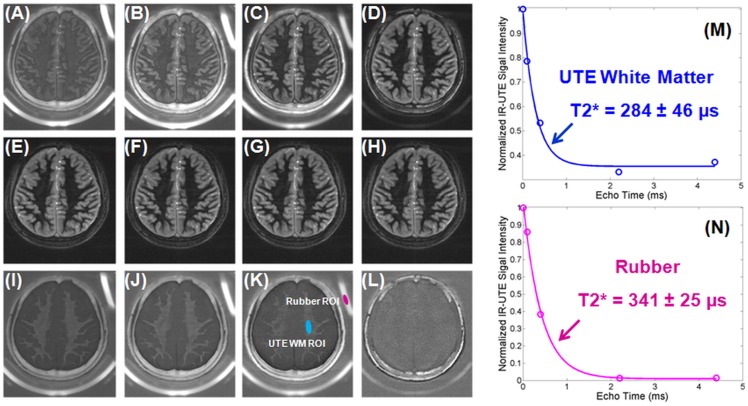
Selected 2D IR-dUTE imaging of a 24 year old healthy volunteer with four sets of dual-echo acquisitions, including TEs of 8 µs (A) and 2.2 ms (E), 0.1 ms (B) and 2.2 ms (F), 0.4 ms (C) and 2.2 ms (G), 2.2 ms (D) and 4.4 ms (H) as well as the corresponding echo subtraction images (I–L). Single component fitting shows a T2* of 284±46 µs for the ultrashort T2* components in white matter of this volunteer. The rubber phantom showed a short T2* of 341±25 µs, which is only a 5.9% overestimate of the true T2* value of 322±9 µs derived from the non-selective UTE acquisitions.

Figure 5 shows 2D IR-dUTE images of the ultrashort T2* components in white matter of the brain acquired with four sets of dual-echo acquisitions. A TI of 420 ms was used together with a TR of 1500 ms. This provided excellent nulling of the long T2 components of white matter as seen by the zero or near zero signal from white matter in the later echo images with TEs of 2.2 or 4.4 ms. Single component exponential signal decay fitting showed a T2* of 284±46 µs for the ultrashort T2* components of white matter, and a T2* of 341±25 µs for the rubber. The rubber T2* was about 5.9% overestimated relative to the reference value of 322±9 µs measured with the 2D non-selective UTE sequence. The small difference suggests that the IR-dUTE sequence provides fast and accurate measurement of T2* of the rubber as well as the ultrashort T2* components in white matter.

**Figure 6:**
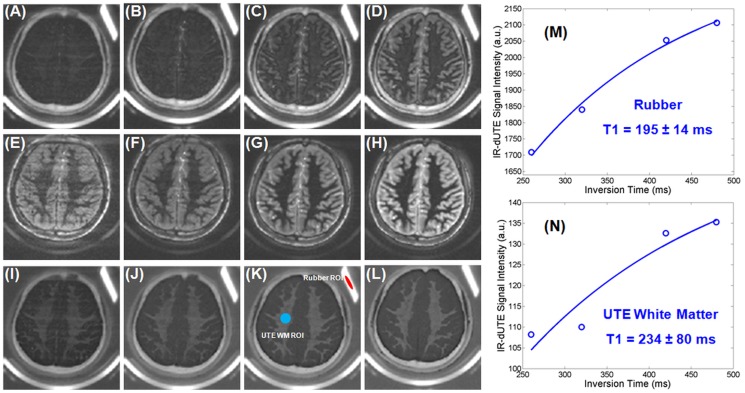
Selected 2D IR-dUTE imaging of a 27 year old healthy volunteer with four different TR/TI combinations: 750/260 ms (A, E, I), 1000/320 ms (B, F, J), 1500/420 ms (C, G, K) and 2000/480 ms (D, H, L). The ultrashort T2* components were depicted with low signal in the first echo images (A–D), and near zero signal in the 2^nd^ echo images (E–H), but highlighted in the echo subtraction images (I–L). Exponential recovery curve fitting shows a short T1 of 195±14 ms for the rubber, which is about a 7% overestimate relative to the reference value of 182 ms derived from the non-selective saturation recovery UTE acquisitions. A short T1 of 234±80 was demonstrated for the ultrashort T2* components in white matter. A significant fitting standard error was observed.

Figure 6 shows IR-dUTE images acquired with four different TR/TI combinations. TI was adjusted for each TR to guarantee that the long T2 components were nulled with zero or near zero signal in the 2^nd^ echo image, as demonstrated by the near noise level SNR of 2.4. Excellent single component exponential recovery curve fitting was achieved for the rubber. A short T1 of 195±14 ms was demonstrated. This T1 was very close to that derived from the non-selective 2D saturation recovery UTE (only 7.1% higher), and the non-selective 2D IR-UTE (only 1.5% lower), suggesting that the IR-dUTE sequence is capable of fast accurate measurement of the T1 of ultrashort T2 species. A short T1 of 234±80 ms was demonstrated for the ultrashort T2* components in white matter of the brain in this healthy volunteer.

High contrast morphological imaging and quantitative evaluation of T2* and T1 were achieved for all six healthy volunteers recruited for this study. The results demonstrate that the ultrashort T2* components in brain white matter have very short T2*s ranging from 0.27 ms to 0.40 ms and short T1s ranging from 197 to 248 ms. On average, a short mean T2* of 0.32±0.09 ms, and a short mean T1 of 226±46 ms were demonstrated for the healthy volunteers at 3T.

## Discussion

The existence of multiple water components in white matter of the brain is well recognized. Multi-component analysis of the CPMG T2 signal decay demonstrates three water components: myelin water (T2∼10–50 ms), intra/extra axonal water (T2∼80 ms) and interstitial water (T2∼2000 ms) [5], [7]. Multi-component T2* analysis of multi-echo gradient echo images suggest that these water components have different T2*s: ∼10 ms for myelin water, ∼38 ms for axonal water and ∼78 ms for interstitial water [24]–[26]. However, the literature regarding T1 relaxation in white matter of the brain is somewhat inconsistent. Some groups reported a uniform T1 for different water components. For example, Whittall et al. investigated the T1 distributions for different white and gray matter structures in the brain using a saturation recovery approach, and found each of the structures was practically mono-exponential [5]. They suggested a single T1 component for both white and gray matter. Breger et al. and MacFall et al. reported similar findings [27], [28]. On the other hand, there are also several groups reporting two or more T1 components in white matter. For example, Koenig et al. and Stanisz et al. suggested two T1 components: a shorter T1 for myelin water, and a longer T1 for other longer T2 components in white matter [4], [6]. Does and Gore reported three components for both T1 and T2 in rat brain and trigeminal nerve in vivo [29]. Lancaster et al. investigated the use of a three-pool relaxation model to measure myelin, myelinated-axon and mixed water pool fractions, and found that the measured water fractions followed values predicted during myelination [30].

However, all of the water components described above are unlikely to be signal sources for the IR-dUTE signal. Conventional clinical sequences with TEs of several milliseconds or longer cannot directly detect signal from the ultrashort T2* components in white matter of the brain, which have T*s of hundreds of microseconds or less [16]. Nayak et al. first studied the ultrashort T2* components in white matter of a fixed cadaveric brain using a long T2 saturated UTE sequence on a clinical 1.5 T scanner, and found T2* values ranged from 100 to 350 µs [31]. Later Larson et al further optimized long T2 suppression pulses for optimized UTE imaging of the short T2 components [32]. However, this approach may have increased long T2 signal contamination compared with the adiabatic inversion recovery approach since saturation based approaches are more sensitive to B1 and B0 inhomogeneities. Waldman et al. first introduced adiabatic inversion recovery prepared 2D UTE imaging of the ultrashort T2* components in white matter of the brain in vivo at 1.5 T [15]. This sequence can potentially detect signal from myelin, which is made up of semi-solid proteins and fatty molecules with extremely short T2*s. Du et al first used IR-UTE sequences to measure the T2*s of the ultrashort T2* components in white matter of the brain using a 3T scanner, and found T2* values of around 300–400 µs [33], which is very close to T2* values of cortical bone [17]. These results suggest that the IR-UTE signal is very different from these detected with conventional clinical sequences. Later Horch et al. further investigated the origins of the UTE signals in myelinated nerve, and suggested the ultrashort T2 signals (50 µs<T2<1 ms) could potentially be used as a specific measure of myelin content [8]. More recently, Wilheml et al. studied the spectrum of myelin in the spinal cord and found that the spectrum could be modeled as a sum of super-Lorentizians with a T2* distribution covering a wide range of values from 8 µs to 26 ms [9]. Both studies found that D_2_O exchange does not affect the signal intensity of the ultrashort T2* components, suggesting that the ultrashort T2* signal of nerve and spinal cord samples were predominantly from myelin lipids.

While it has been confirmed that there are ultrashort T2* components in white matter of the brain with extremely short T2* values, the T1s of these components have not been well studied. As far as the authors know, the only published T1 measurement was done by Nayak et al., who employed a 4 to 8 ms nonselective hard 90° excitation pulse together with a dephaser to suppress the long T2 magnetization, followed by UTE acquisitions (with minimal nominal TEs of 228 µs) at a series of TRs (100, 300 and 1000 ms) to measure T1 of the ultrashort T2* components in white matter in fixed cadaveric brain [31]. They found a majority of the signal was from T1s in the range of 300–500 ms. There are several problems associated with this approach. First, the T1 of white matter in fixed brain may be very different from that in vivo. Formalin fixation may significantly reduce T1 of the long T2 components in white matter (our recent unpublished results suggest that formalin fixation can reduce T1 to as short as ∼300 ms). T1 of the ultrashort T2* components may also be affected by formalin fixation. Second, it is difficult to completely saturate the long and medium T2 components using a single long duration square pulse with a narrow spectral bandwidth [17]. Significant residual long T2 signal may be detected due to both B1 and B0 inhomogeneities. This residual signal may be very significant relative to the size of the signal from the ultrashort T2* components in white matter that have very low proton densities. Third, the UTE data acquisition started after a long duration 90° saturation pulse, and was followed by gradient dephasing. There may be a significant delay between the center of the saturation pulse and the excitation pulse (typically >10 ms). Long T2 signal recovery during this period will produce a bias in UTE imaging of the ultrashort T2* components. This signal bias may be significant considering the low proton densities of the ultrashort T2* components. Fourth, the saturation recovery approach would work more robustly for more uniform short T2 tissues such as cortical bone, where short T2* and short T1 values are expected across the whole cortical cortex. The white matter contains a mixture of both short and long T2 tissue components, with the majority being the long T2 components. This puts a very high premium on efficient long T2 signal suppression in T1 measurement studies.

The novel approach proposed in this paper appears to be a significant improvement. First, it is based on the IR-dUTE sequence, where the adiabatic IR pulse provides efficient long T2 inversion and signal nulling. The detected IR-dUTE signal is selective for the ultrashort T2* components, as evidenced by the excellent single component T2* decay behavior (Figure 5). Second, the adiabatic IR pulse is non-slice selective with a relatively broad spectral bandwidth of 1.4 kHz, which minimizes sensitivity to both B0 and B1 inhomogeneities assuming the adiabatic condition is met. Third, the ultrashort T2* components have T2*s of around 300–500 µs, which means the adiabatic IR pulse nearly completely saturates their longitudinal magnetizations (i.e., Q∼0). T1 quantification can be simplified to a single exponential recovery as described by Eq. 5. Since each TR/TI combination was carefully chosen to guarantee excellent nulling of the long T2 components (judged by near zero signal in the 2^nd^ echo image), the characterized signal is believed to be selective for the ultrashort T2* components in white matter. As a result, a simplified T1 measurement can be achieved. This approach may also allow accurate measurement of T1 of other ultrashort T2* species such as cortical bone. We compared T1 measurement of a piece of bovine cortical bone using the two approaches described above, and found the IR-UTE approach provided a T1 of 266±9 ms which is very close to the T1 of 253±18 ms produced by the standard saturation recovery approach (Figure 3).

However, it is still unclear whether the IR-dUTE signal is selectively from myelin, water tightly bound to myelin, or a combination of myelin and water tightly bound to myelin. The macromolecular pool of lipid protons trapped within the myelin sheaths may have a spectrum of T2. These with T2 around or less than 50 µs are not detectable with 2D IR-UTE sequences on clinical MR systems, even though a minimal nominal TE of 8 µs is used. However, some lipid protons oriented near the magic angle (∼55° relative to the B0 field) may have prolonged T2 and thus become detectable with clinical UTE sequences. Some lipid protons with T2 in the range of a few hundred microseconds (e.g., T2*∼250 µs in frog sciatic nerve, T2*∼700 µs in rat optic nerve) are clinically detectable with UTE sequences [8]. Water tightly bound to myelin may have a relatively short T2* and may not be completely nulled by the adiabatic IR pulse. Furthermore, it is difficult to achieve perfect nulling of the long and medium T2 water components in white matter. Some studies suggest that these water components may have different T1s [4], [6], [29], [30], and thus cannot be nulled by a single IR pulse. As a result, we suspect that the IR-dUTE signal source may include both myelin and water tightly bound to myelin. D_2_O-exchange studies may help clarify the signal source of IR-dUTE imaging of the ultrashort T2* components.

There are several limitations of this study. Most notably the signal recovery curve shown in Figure 6N has a relatively large standard error in exponential curve fitting. There are several explanations for this behavior. First, the ultrashort T2* components in white matter have very low proton densities, which results in an inherently low SNR. Second, the half projections were significantly undersampled in order to reduce the total scan time. Only 131 projections were sampled for a readout of 191, which corresponds to an undersampling factor of 2.3. The undersampling streak artifacts may distort the signal recovery curve. Noise correction based on the Miller approach may only partially resolve issues associated with low SNR and undersampling streak artifacts, where accurate measurement of noise is challenging. Third, a single T1 was assumed for the long T2 components in white matter of the brain. It is difficult to null all the long T2 components with a single IR pulse if there is more than one T1 component [4], [6], [29]. All the factors above may explain why the IR-UTE approach perfectly describes the exponential recovery curve for rubber phantom which has a single T1 with relatively high proton density (∼44%, unpublished results) and SNR, than it does the ultrashort T2* components in white matter of the brain. Fourth, the effects of possible exchange between different pools of protons are not considered in T1 measurement. Recent work by Ou et al shows that MT effects may lead to more than 10% errors in T1 for the long T2 components in white matter [34]. More research is needed to investigate how these effects affect T1 for the ultrashort T2* components in white matter of the brain.

The relative contributions of myelin water protons (if any) and non-water protons to the IR-dUTE signal will be investigated in future studies. The IR-dUTE sequence is a 2D technique and is subject to eddy currents. 3D IR-dUTE imaging based on radial or cone trajectories provides volumetric coverage with less sensitivity to eddy currents [18], [35], and will be investigated in future studies. Future work will also include study of patients with MS and investigate the quantitative changes in T1, T2* and proton densities of the ultrashort T2* components in white matter of the brain with myelin degeneration/regeneration. These techniques may provide novel approaches for diagnosis as well as therapeutic monitoring in MS.
